# Clinical and Bronchoscopy Assessment in Diagnosing the Histopathology Type of Primary Central Lung Tumors

**DOI:** 10.2174/0118743064318977240531100045

**Published:** 2024-06-06

**Authors:** Mia Elhidsi, Jamal Zaini, Lisnawati Rachmadi, Asmarinah Asmarinah, Aria Kekalih, Noni Soeroso, Menaldi Rasmin

**Affiliations:** 1 Department of Pulmonology and Respiratory Medicine, Faculty of Medicine, Universitas Indonesia, Indonesia; 2 Department of Anatomic Pathology, Faculty of Medicine, Universitas Indonesia, Indonesia; 3 Department of Biomedical Sciences, Faculty of Medicine, Universitas Indonesia, Indonesia; 4 Community Medicine, Faculty of Medicine, Universitas Indonesia, Indonesia; 5 Pulmonology and Respiratory Medicine, Faculty of Medicine, University of North Sumatra, Medan,Indonesia

**Keywords:** Bronchoscopy, Clinical assessment, Diagnosis, Histopathology, Lung tumors, Primary central lung tumors

## Abstract

**Background:**

The location and type of a tumor influence the prognosis of lung cancer. Primary Central Lung Tumors (PCLTs) are correlated with poor prognoses and certain histologic types. This study aimed to present a comprehensive exploration of clinical and bronchoscopic assessments for diagnosing the histopathology types of PCLTs and identified the factors associated with certain histologic types.

**Methods:**

This was an observational cross-sectional study of PCLTs, defined as tumors in direct contact with hilar structures or located within the inner two-thirds of the hemithorax. We gathered demographic and clinical data, as well as data on bronchoscopy assessment and histopathology type. Tumor stage, symptoms of superior vena cava syndrome, and enlargement of lymph nodes in the paratracheal and subcarinal regions were also documented.

**Results:**

Of the 895 patients, 37.87% had primary lung tumors, with 17.76% classified as PCLTs. Notably, PCLT cases exhibited a higher proportion of stage III (28.9% *vs*. 18.3%; *p* = 0.03) and Squamous Cell Carcinoma (SCC) histopathology (37.1% *vs*. 17.2%; *p* = 0.00) compared with non-PCLT cases. Bronchoscopic findings in PCLTs revealed a predilection for central airway masses (25.2%) and compressive distal airway stenosis (25.2%). Subgroup analysis of 159 PCLT cases identified 37.10% as SCC. Multivariate analysis underscored that intraluminal masses predict central SCC (odds ratio 2.075, 95% confidence interval 1.07–3.99; *p* = 0.028).

**Conclusion:**

The proportion of stage III, SCC histopathological type, and intraluminal lesions was higher in patients with PCLT than in non-PCLT cases. The presence of intraluminal lesions can predict the histopathological type of SCC in patients with PCLTs.

## 1 INTRODUCTION

The landscape of lung cancer management is continually advancing. Nevertheless, lung cancer persists as a global health concern, with substantial mortality and morbidity implications. As the foremost cause of cancer-related deaths worldwide, lung cancer poses a substantial burden on both men and women alike [1]. The prognosis of lung cancer depends on various factors, such as histopathological type, staging, molecular biology, performance status, and, notably, tumor location. The location of the tumor notably influences the prognosis of lung cancer. Lung tumors characterized by central lesions are correlated with a less favorable prognosis because of their tendency for intrathoracic dissemination and complications arising from tumor mass compression within the mediastinal cavity and chest wall [2, 3]. However, consensus on the classification of central lung tumors varies across studies, contributing to a lack of uniformity in defining these lesions. According to the American College of Chest Physicians guidelines, a lung tumor is deemed centrally located if it is situated within one-third of the inner hemithorax. By contrast, the National Comprehensive Cancer Network and the European Society of Thoracic Surgery categorize central tumors as those within the inner two-thirds of the hemithorax. Furthermore, radiation oncologists define central lung tumors as those positioned two centimeters from the proximal bronchus, the heart, major blood vessels, trachea, and other mediastinal structures. This particular definition serves as a benchmark for comparing the safety profiles of radiation therapy administration between central and peripheral tumors [4-6]. In this context, bronchoscopy, which is the standard examination for respiratory tract lesions, can also be utilized as a basis for the more accurate identification of central lung lesions.

The attributes of Primary Central Lung Tumors (PCLTs) featuring central lesions have undergone thorough investigation, encompassing demographic characteristics, clinical symptoms, staging, complications, and, notably, histopathological type. In the context of stem cell carcinogenesis, lung cancer with a central lesion tends to be associated with Squamous Cell Carcinoma (SCC) and small cell carcinoma, while adenocarcinoma is more commonly found in peripheral lesions [7-10]. However, recent studies have reported a higher incidence of SCC in peripheral locations [11]. Nevertheless, SCC is still regarded as having distinct characteristics in PCLT compared with peripheral tumors [12]. Additionally, PCLTs need comprehensive examination owing to their high risk of spreading, particularly to the lymph nodes, and their association with an unfavorable prognosis [13].

In this context, bronchoscopy emerges as the standard examination for respiratory tract lesions and serves as a valuable tool for the accurate identification of PCLTs. The bronchoscopic findings in PCLTs often present as compression, intraluminal masses, or a combination of both, further emphasizing its diagnostic significance in this subset of tumors. The location of the lesion is a pivotal factor influencing the success of diagnostic bronchoscopy. Overall, the diagnostic yield of bronchoscopy biopsy ranges from 55% to 83%. Diagnostic bronchoscopy for central lesions demonstrates higher accuracy than that for peripheral lesions [5]. Furthermore, Transbronchial Needle Aspiration (TBNA) biopsy has emerged as a reasonably accurate diagnostic modality for central lung tumors, whether conventional or ultrasound-guided [14-16]. Their elevated risk of metastasis to mediastinal lymph nodes adds complexity to the prognosis, subsequent management decisions, and diagnostic approaches [17, 18]. Early and accurate diagnosis is crucial for selecting appropriate therapy, thus improving prognosis and enhancing patients’ quality of life.

The primary outcome of this study was the evaluation of the clinical characteristics of PCLTs, their bronchoscopy assessment, and the histopathology types of PCLTs. The secondary outcome was the identification of factors associated with central squamous lung tumors.

## 2 MATERIALS AND METHODS

### 2.1 Study Population and Data Collection

**Fig. (1):**
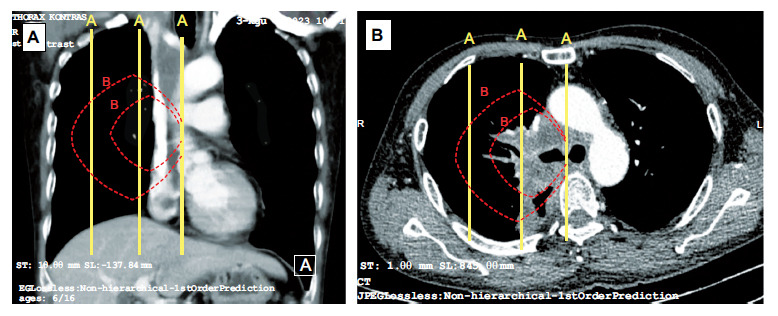
The demarcation lines for dividing the hemithorax into three-thirds using straight lines (**A** lines) and concentric lines (**B** lines). (**A**). CT image in the axial plane. (**B**). CT image in the coronal plane.

This observational, cross-sectional, analytical study was conducted at Persahabatan Hospital in Jakarta, Indonesia, from March 2023 to October 2023. The study protocol was approved by the Persahabatan Hospital Institutional Review Board with ethical clearance number 13/KEPK-RSUPP/01/2023. The eligible participants were patients aged 18 years and above undergoing elective diagnostic bronchoscopy procedures for suspected PCLTs. The exclusion criteria were patients facing immediate life-threatening central airway obstruction, those with nonprimary lung tumors, those with mediastinal tumors, those with histopathological findings other than lung cancer, and participants who did not provide informed consent. Primary central lung tumors were determined using radiographic chest imaging and thoracic computed tomography (CT) scans. A central lesion was operationally defined as a tumor in direct contact with hilar structures (*i.e*., lobar bronchi, lobar or main pulmonary arteries, or main pulmonary veins) or located within the inner two-thirds of the hemithorax [19]. The demarcation lines for dividing the hemithorax into three-thirds were established using straight lines in the sagittal plane or concentric lines originating in the hilum (Fig. **1**). The calculations for these divisions were based on chest CT taken at least two weeks before the procedure. The evaluation was conducted through consensus between two radiologists.

We gathered demographic and clinical data, including age, gender, chief complaints, smoking history, cancer history, bronchoscopy assessment, and histopathology type. Tumor stage, symptoms of Superior Vena Cava Syndrome (SVCS), and enlargement of lymph nodes in the paratracheal and subcarinal regions were also documented. The elderly are defined as individuals aged > 65 years [20]. Smokers were individuals with a minimum smoking history of 20-pack-years [21]. A history of malignancy refers to any prior malignancy other than lung cancer that is considered cured or in remission without ongoing treatment. Lung cancer staging was classified into resectable stages I–II, stage III without distant metastasis, and stage IV with distant metastasis. Superior vena cava syndrome was identified by an elevation in vascular pressure in the upper body arising from the obstruction of the superior vena cava. Clinically, it is characterized by symptoms such as dyspnea, cough, facial and neck swelling, and other symptoms and signs contingent upon the severity of the obstruction. Superior vena cava syndrome was defined as SVCS grade ≥ 2, indicating a moderate degree characterized by edema in the head or neck accompanied by functional impairment [22].

### 2.2 Bronchoscopy Assessment

Bronchoscopy procedures in this study were employed to assess the condition of the airways and perform a lung biopsy. Before undergoing bronchoscopy, all patients pro-vided written consent, underwent a cardiology examina-tion, and fasted for a minimum of 6 hours. Bronchoscopy preparation, tolerance assessment, contraindication eva-luation, and other bronchoscopy procedures were conducted according to the standard operating procedures (SOPs) at Persahabatan Hospital. These bronchoscopy procedures were performed by pulmono- logists with a minimum of five years of experience in conducting bronchoscopies. The bronchoscope utilized was the Fuji bronchoscope with the EB580S or EB580T scope. Anesthe-sia during bronchoscopy involves intravenous sedation with general anesthesia or local anesthesia with mild to moderate sedation. A laryngeal mask airway was emplo-yed for procedures under moderate general anesthesia, while a scope was inserted through the mouth for topical anesthesia. Hemodynamic monitoring and oxygen saturation were continuously monitored throughout the procedure.

The bronchoscopy examination was initiated from the vocal cords, progressing through the trachea, the right main bronchus and its branches, and the left main bronchus and its branches. We documented the entire bronchoscopy procedure through photographs and video recordings of the intervention. For intraluminal masses, forceps biopsy was performed utilizing the Radial Jaw 4 Pulmonary Single-Use Biopsy Forceps. Forceps biopsy was performed with a minimum of three sampling attempts. Further investigation of intraluminal masses involved biopsy brushing using the Cellebrity™ Single-Use Cytology Brush Boston. In cases in which intraluminal masses were not present, a transbronchial biopsy was performed using forceps or a brush, guided by fluoroscopy, radial Endobronchial Ultrasound (EBUS), or a combination of both. The transbronchial biopsy involved a minimum of five sampling attempts. Post-biopsy bleeding was managed according to the hospital’s SOPs. Tissue samples obtained *via* forceps were then fixed in formalin, while those collected through brushing were smeared on glass slides and subsequently fixed in 96% alcohol solution. Bronchial lavage was uniformly performed on all participants undergoing bronchoscopy. The tissue biopsies obtained were further processed for histological examination, while the smears from brushing and bronchial washing were continued for cytological examination.

The involvement of mediastinal lymph node stations paratracheal 4R, 4L, and subcarina 7 was accomplished through EBUS or assessment based on radiology [23]. In some patients, a conventional transbronchial needle aspiration biopsy was performed. Smears were made on glass slides of the TBNA specimen, followed by a cytological examination. The enlargement of mediastinal lymph nodes was determined by measuring the diameter (1cm, according to chest CT or EBUS findings [24-26]). Data extracted from bronchoscopy included visual observations of intraluminal lesions, such as mass, external compression, and normal airways. Additionally, detailed documentation was made regarding the bronchoscopy modalities employed in each procedure, such as forceps biopsy, brushing, and transbronchial needle aspiration. The diagnostic yield of each modality was calculated by determining the number of procedures that resulted in a specific pathological diagnosis divided by the total number of procedures conducted [27].

### 2.3 Histopathology Examination

The histopathological examination was conducted at the pathology laboratory of Persahabatan Hospital. The examination of anatomic pathology utilizing paraffin blocks and Hematoxylin–Eosin (HE) staining encompassed several critical stages. The process commenced with tissue processing using a tissue processor machine, in which tissues underwent dehydration, clearing, and embedding stages. Subsequently, paraffin blocks were crafted by placing the processed tissues into molds along with liquid paraffin. Upon solidification, the paraffin blocks were sectioned into thin slices using a microtome.

The resultant paraffin sections were then stained with HE dye following a series of steps involving drying and adherence, rehydration to remove paraffin, staining of the nuclei and cytoplasm, and dehydration and clearing. Subsequently, the tissue sections melted the paraffin to adhere to the glass slides, ready to be cut and stained. Finally, the stained and prepared tissue sections underwent examination and diagnosis by consensus between at least two specialists in anatomic pathology. The histopathological types of the final diagnoses were categorized based on the World Health Organization 2021 classification [28]. An immunohistochemical examination was not included in the operating procedures for this study.

### 2.4 Data Analysis

The descriptive analysis of categorical variables involved presenting percentages, while numeric variables were expressed as either means or medians. The identification of disparities in characteristics between central and non-central lesions employed statistical tools, such as the chi-square test, with alternative analyses, such as Fisher’s exact test, implemented when the cell count was below 5. Significant differences between proportions were established if the *p*-value was < 0.05, with a 95% confidence interval (CI).

In a subgroup PCLT analysis, we further explored the differences in central lesion characteristics between squamous and non-squamous histopathology. We conducted bivariate and multivariate analyses to explore the factors related to squamous histopathology within the PCLT population, employing logistic regression. Variables with a *p*-value < 0.25 in the bivariate analysis were included in the multivariate analysis. The risk magnitude was expressed as odds ratios (ORs) with a 95% CI and considered significant if the *p*-value was < 0.05. Data processing was carried out using IBM SPSS Statistics 25.0.

## 3 RESULTS

**Fig. (2):**
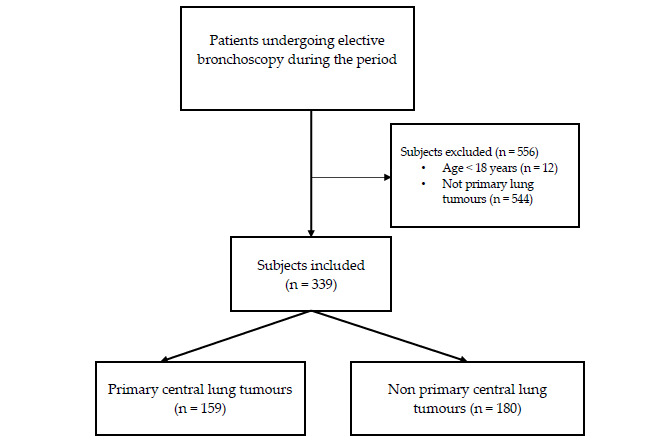
Flowchart of study design.

A total of 895 participants underwent bronchoscopy during the study period of March 2023–October 2023; of the tumors, 37.87% were primary lung tumors, and 17.76% were PCLTs. Five hundred and fifty-six participants were excluded from the study for reasons such as being children, having mediastinal tumors, or having nonprimary lung tumors. The analysis included a final sample of 339 participants, including 159 PCLTs and 180 non-PCLTs (Fig. **2**). A subgroup analysis was then conducted on 159 PCLT patients.

Both PCLT and non-PCLT groups demonstrated comparable median ages (60 *vs*. 61 years old, *p* = 0.357) with no significant variations noted in gender distribution (male 71.7% *vs*. 72.5%, *p* = 1.000), elderly age representation (27.7% *vs*. 32.2%, *p* = 0.428), or smoking prevalence (69.2% *vs*. 66.7%, *p* = 0.705) between the two cohorts. The primary complaint of cough occurred slightly more frequently in the PCLT group than in the non-PCLT group (37.1% *vs*. 27.8%, *p* = 0.086). The incidence of chest pain was higher in the PCLT group than in the non-PCLT group, although the difference was not statistically significant (22.6% *vs*. 18.9%, *p* = 0.473). A higher proportion of stage III cases was noted in the PCLT group than in the non-PCLT group (28.9% *vs*. 18.3%; *p* = 0.03). The involvement of paratracheal and subcarinal lymph nodes was more prevalent in the PCLT group than in the non-PCLT group (69.8% *vs*. 57.8%; *p* = 0.029). All histopathological types of lung cancer in this study were diagnosed based on HE staining examination agreed upon by two specialist anatomical pathologists; further immunohistochemical examination was not required. Although adenocarcinoma remained the most prevalent lung tumor type, its proportion in PCLTs was lower than in non-PCLTs (53.5% *vs*. 76.1%; *p* = 0.00). The most common histopathological types in the PCLT group were adenocarcinoma (53.5%), SCC (37.1%), and small cell carcinoma (6.3%). Squamous cell carcinoma histo- pathology was more common in the PCLT group than in the non-PCLT group (37.1% *vs*. 17.2%; *p* = 0.00), although the proportion of SVCS was higher in PCLTs than in non-PCLTs (12.6% *vs*. 9.4%, *p* = 0.454) (Table **1**).

In general, bronchoscopy in primary lung tumors revealed airway involvement, either intraluminal lesions or luminal compression, with only 14.2% showing normal airways. The overall diagnostic yield of bronchoscopy in primary lung tumors was quite good, with the highest diagnostic yield seen in forceps biopsy (87.9%) and the lowest seen in transbronchial biopsy (73.7%). Bronchoscopy findings in PCLTs were more likely to involve central airway intraluminal masses (25.2%), followed by compressive distal airway stenosis (25.2%). A notable 8.2% of the participants presented with normal airways despite radiological evidence of a central mass. The proportion of central intraluminal masses was higher in the PCLT group, while the proportion of normal airways was higher in the non-PCLT group. The diagnostic yield of bronchoscopy for lung tumors reached 73.7%–87.9%. The diagnostic accuracy of bronchoscopy for PCLT was lowest with TBNA at 69.0% and highest with forceps biopsy at 86.6%. For the non-PCLT group, the lowest diagnostic accuracy of bronchoscopy was with brushing, which was at 72.4%, and the highest was with forceps, which was at 89.2% (Table **2**).

In a subgroup analysis of 159 PCLT cases, 37.10% were identified as SCC. There were no significant differences in gender proportions, smoking status, primary complaints, malignancy staging history, or SVCS incidence between central SCC and central non-SCC. The proportion of elderly patients in the SCC group was higher than that in the non-SCC group (33.9% *vs*. 24.0%; *p* = 0.023). The proportion of smokers in the non-SCC group was higher than that in the SCC group, although it was statistically nonsignificant (62% *vs*. 38.2%; *p* = 0.808). Central SCC also tended to have intraluminal masses compared with non-SCC (61.0% *vs*. 43.0%; *p* = 0.042) (Table **3**). In the bivariate analysis results, we found that only intraluminal lesions were associated with central SCC. Multivariate analyses of the factors related to central SCC revealed that intraluminal masses could predict central SCC, with an OR of 2.075 (95% CI OR 1.07–3.99; *p* = 0.028) (Table **4**).

## 4 DISCUSSION

Our study reported the clinical characteristics of PCLTs that were predominantly identified in males with a history of smoking in their sixth decade in stage 4, with an adenocarcinoma histopathological type. We also confirmed the predictive factors for squamous central lung cancer. Until now, histopathological examination has remained the gold standard for diagnosing lung tumors and determining the specific cell type of the tumor [29]. We found a higher prevalence of PCLTs with the inner two-thirds thorax criteria than Choi *et al*.’s study, which reported a 29.8% prevalence of central lung cancer. The prevalence further decreased to 12.6%, with a narrower criterion for the concentric inner one-third from the midline [14]. The age and male proportion of the sample in our PCLT study were relatively younger and lower, respectively, compared with those in some previous studies [10, 30]. Although the PCLT location was in the midline, the proportion of SVCS in our study was not higher than that in the non-PCLT cases. Malignancies involving the mediastinal area are responsible for over two-thirds of SVCS cases and can be early manifestations of a previously undiagnosed malignancy. The prevalence of SVCS reported in the literature ranged from 1 in 650 patients to 1 in 3,100 patients. Our study reported a higher prevalence than previously documented in both the overall primary lung tumor group and the PCLT subgroup.

This study revealed a higher prevalence of stage III cases in PCLTs compared to non-PCLTs. Stage III, characterized as a locally advanced stage, offers a diverse range of therapeutic options, encompassing surgery, various systemic treatments, and radiotherapy, often in combination. The factors influencing the consideration and prognosis of stage III include tumor size, paratracheal and subcarinal lymph nodal involvement, and local infiltration, in addition to patient tolerance to different therapeutic options. While some cases aim for curative therapy, a significant proportion adopts a palliative approach because of the persistently low survival rates in stage III, ranging from 13% to 36%, with a median overall survival spanning from 9 to 34 months [31-33].

In stage III PCLT, in which distant metastases are absent, mediastinal lymph node involvement emerges as the most crucial prognostic factor, necessitating a proper assessment of these nodes. Previous studies have reported that in approximately one-third of patients, metastatic tumor cells spread directly to the mediastinal lymph nodes, bypassing the hilar lymph nodes [34]. The involvement of paratracheal and subcarinal lymph nodes in this study was notably high and could potentially be even higher with a screening biopsy in all cases. Furthermore, this study utilized the criterion of two-thirds within the hemithorax as the definition of central lesions, while Shin’s study suggested that lesions occupying one-third of the hemithorax are already associated with occult mediastinal lymph node metastasis [35]. Other diagnostic modalities, such as Positron Emission Tomography (PET) scans, can also be utilized to assess the involvement of mediastinal lymph nodes. While this examination is less invasive, it is also more costly than a biopsy. Several studies have indicated that PET scans may not always distinguish between malignant and benign processes, as confirmed by histopathological tissue examinations. Negative PET scan results are generally effective in ruling out malignancy, but positive PET scans may be attributable to malignancy, inflammation, or other reactive conditions. Therefore, lymph node biopsy remains recommended for the final diagnosis and for determining pathological type [36-38]. In the context of PCLT, mediastinal lymph node involvement can serve as a diagnostic modality not only for confirming staging, especially in cases of potentially resectable tumors, but also for determining the histopathological types of PCLTs [39, 40]. Although TBNA has advanced with EBUS and rapid onsite evaluation, conventional TBNA is still practiced by Indonesian pulmonologists, particularly for stations 4R and 7. Despite the small sample size in this study, the accuracy of conventional TBNA was exceptionally high, comparable to earlier studies reporting diagnostic yields in benign cases ranging from 43% to 93.7% [41].

The evaluation of mediastinal lymph nodes should be an integral part of bronchoscopy procedures, and biopsies, even those using conventional TBNA, should be conducted. Utilizing specialized combinations of bronchoscopy modalities, particularly biopsy, and EBUS-TBNA, has the potential to further enhance diagnostic yield. The features observed during bronchoscopy can also assist in choosing appropriate diagnostic bronchoscopy modalities. Previous studies have demonstrated that the diagnostic yield of bronchoscopy for central lesions is approximately 73%–81%. These results indicate a higher diagnostic yield for central lesions compared with peripheral lesions. Compared with biopsy sampling of non-intraluminal lesions, that of intraluminal lesions is relatively easier to perform using forceps biopsy and brushing without the assistance of guiding modalities [42].

Adenocarcinoma still constitutes the majority of the histopathological types in lung cancer, followed by SCC, including the PCLT group in this study [43]. Although recent studies have reported a higher prevalence of SCC in non-PCLT lesions, our study indicates that there is still a significant proportion of SCC in the PCLT group [44, 45]. Several similar studies have also noted varying distributions of histopathological types between PCLT and non-PCLT lesions. Studies by Mizushima *et al*. and William Krimsky *et al*. revealed variations in tumor location distribution [11, 46]. Mizushima *et al*. reported that out of 235 SCC cases, 129 were peripheral, and 106 were central. By contrast, William Krimsky *et al*. found that among 56% of SCC patients, 55% had peripheral and 45% had central locations [11]. Divergent results were noted by Dongiun *et al*., who stated that out of 142 studied SCC lesions, a majority (121 out of 142) were in central regions, with a significant proportion affecting the mediastinum [47].

Furthermore, this study found that bronchoscopy assessment of intraluminal lesions could predict the presence of SCC in PCLTs. Bronchoscopy, with its intrabronchial imaging capabilities, is expected to be superior to radiological imaging in predicting histopathological types, although further research is needed to confirm this [48-50]. Our findings align with several previous studies showing that SCC mostly manifests as an endoluminal lesion in the central airway, posing a high risk of bleeding during biopsy [51, 52]. Furthermore, Sueqi *et al*. highlighted that SCC often involves the main bronchus, frequently necessitating sleeve resection in its management [13]. Morphologically, SCC originates from metaplasia pathology, progressing through dysplasia to become SCC, which not only infiltrates the airways but also poses a risk of central airway obstruction, sometimes becoming life-threatening [53]. The ablation of central airway lesions with various modalities may be necessary in such conditions [54-56]. Despite the demographic and clinical characteristics in our study not being predictive of SCC histopathology, other studies suggest that SCC, especially with intraluminal lesions, is associated with symptoms such as cough, dyspnea, hemoptysis, and obstructive syndrome [57, 58]. A smoking history of more than 30 pack years, as reported by Ban *et al*., was associated with the male gender and a high proportion of SCC [59]. However, Zeng *et al*. reported that among smokers, the proportion of SCC decreased from 40.5% to 23.7% over a period of 13 years [60].

This study, conducted as a single-center observational investigation at Persahabatan Hospital in Jakarta, Indonesia, presents noteworthy findings while also exhibiting inherent limitations. Initially, the single-center design of the study, which was conducted at Persahabatan Hospital, a national referral center and educational institution, raises concerns regarding the generalizability of the findings to broader populations. Given the potential variations in clinical practices and patient characteristics across different locations, caution should be exercised in extrapolating these findings to diverse healthcare settings. Further multi-center research endeavors are essential to validate and enhance the external applicability of the study’s outcomes. Advanced bronchoscopy techniques, such as electromagnetic and virtual bronchoscopic navigation, are not yet widely adopted in our center.

Additionally, reliance on elective bronchoscopy procedures may introduce selection bias, influencing the representation of cases with varying severity levels. Variations in bronchoscopy biopsy techniques could also affect diagnostic success rates, and the subjective nature of some evaluation parameters underscores the need for careful consideration of potential measurement errors. Although the study provides valuable insights, it is crucial to interpret the results with an awareness of these methodological constraints and to recognize the imperative for future research to address these limitations comprehensively.

Despite its limitations, this study is notable for its comprehensive examination of the clinical characteristics, histopathology, bronchoscopic findings, and diagnostic modalities in PCLTs. This cross-sectional study involves a large number of subjects and primary data collected, thus reducing the bias of missing or invalid data. A significant strength lies in the thorough analysis of factors associated with SCC histopathology within the PCLT subgroup utilizing both bivariate and multivariate methods. Although we investigated various demographic variables and potential factors related to the differing histopathological distribution between PCLTs and non-PCLTs, we could not identify a specific cause for this shift. We propose conducting more extensive prospective studies to explore the factors driving this shift comprehensively. This thorough examination deepens our comprehension of the intricate nature of PCLTs, specifically illuminating the prevalence and characteristics of SCC. Such a targeted and rigorous strategy offers valuable perspectives for clinicians and researchers engaged in lung tumor research.

## CONCLUSION

Primary central lung tumors were predominantly identified in males with a history of smoking in their sixth decade and were commonly found in the advanced stage with an adenocarcinoma histopathological type. The proportion of stage III, SCC histopathological type, and intraluminal lesions was higher among patients with PCLTs than among non-PCLT cases. The presence of intraluminal lesions can predict the histopathological type of SCC in patients with PCLTs.

## AUTHORS’ CONTRIBUTIONS

Conceptualisation, all the authors; methodology, all authors; software, ME, AK; validation, ME, JZ, LR; funding, all the authors; writing, ME, JZ, NNS; writing review, ME, JZ, LR; data analysis, ME, AK; investigation, ME, JZ, LR, AK; supervision, MR, AS, NNS. All authors have read and agreed to the published version of the manuscript.

## Figures and Tables

**Table 1:** Demographic and clinical characteristics of primary central lung tumours (n = 339).

**Variables**	**PCLT** **n = 159** **n (%)**	**Non-PCLT** **n = 180** **n (%)**	**Overall** **n = 339** **n (%)**	***p*-value**
Male	114 (71.7)	130 (72.5)	244 (72.0)	1.000
Age (median, in year)	60 (26-84)	61 (25-82)	60 (25-84)	0,357
Elderly	44 (27.7)	58 (32.2)	102 (30.)	0.428
Smokers	110 (69.2)	120 (66.7)	230 (67.8)	0.705
Chief Complaint Dyspnoea Cough Haemoptysis Chest pain	53 (33.3) 59 (37.1) 11 (6.9) 36 (22.6)	79 (43,9) 50 (27.8) 16 (8.9) 35 (19.4)	132 (38.9) 109 (32.2) 27 (8.0) 71 (20.9)	0.060 0.086 0.640 0.473
History of Malignancy	7 (4.4)	4 (2.2)	11 (3.2)	0.410
Staging of lung tumour Ia–IIb IIIa–IIIc IVa–IVc	6 (3.8) 46 (28.9) 107 (67.3)	10 (5.6) 33 (18.3) 137 (76.1)	16 (4.7) 79 (23.3) 244 (72.0)	0.606 0.030* 0.092
Paratracheal and subcarinal lymphadenopathy	111 (69.8)	104 (57.8)	215 (63.4)	0.029*
Histopathology Squamous cell carcinoma Adenocarcinoma Neuroendocrine carcinoma Small cell carcinoma Cystic adenoid carcinoma	59 (37.1) 85 (53.5) 3 (1.9) 10 (6.3) 2 (1.3)	31 (17.2) 137 (76.1) 1 (0.6) 10 (5.6) 1 (0.6)	90 (100) 222 (100) 4 (1.2) 20 (5.9) 3 (0.9)	0.000* 0.000* 0.345 0.956 0.602
SVCS	20 (12.6)	17 (9.4)	37 (10.9)	0.454

**Abbreviations: **PCLT, primary central lung tumours; SVCS, Superior vena cava syndrome.

*Statistically significant.

**Table 2:** Bronchoscopy assessment of primary central lung tumours.

Bronchoscopy Assessment	**PCLT** **n = 159** **n (%)**	**Non-PCLT** **n = 180** **n (%)**	**Total** **n = 339**	***p*-value**
Bronchoscopy findings, n (%) Intraluminal lesion Compressive stenosis Normal	79 (49.7) 67 (42.1) 13 (8.2)	69 (38.3) 76 (42.2) 35 (19.4)	148 (43.7) 143 (42.2) 48 (14.2)	0.046* 1.000 0.005*
Bronchoscopy diagnostic yield, n/n (%) Forceps biopsy Brushing Conventional TBNA Transbronchial biopsy	58/67 (86.6) 18/22 (81.8) 41/49 (69.0) 26/38 (83.6)	66/74 (89.2) 21/29 (72.4) 17/22 (77.3) 47/61 (77)	124/141(87.9) 39/51 (76.5) 58/71 (81.7) 73/99 (73.7)	0.827 0.652 0.524 0.475

**Abbreviations: **PCLT, primary central lung tumours; TBNA, transbronchial needle aspiration.

*Statistically significant.

**Table 3:** Clinical characteristics of squamous central lung tumours.

**Characteristics**	**Squamous** **n = 59** **n (%)**	**Non-Squamous** **n = 100** **n (%)**	**OR** **(95% CI)**	***p-value***
Male	44 (74.6)	70 (70,0)	1.25 (0.61 – 2.59)	0.662
Elderly	20 (33.9)	24 (24,0)	1.62 (0.8 – 3.29)	0.244
Smokers	42 (38.2)	62 (62,0)	1.16 (0.57 – 2.35)	0.808
Dyspnoea as the main complaint	21 (35.6)	32 (32,0)	1.17 (0.50 – 2.31)	0.772
Advanced stage	49 (83.1)	86 (86,0)	0.79 (0.33 – 1.93)	0.785
SVCS	7 (11.9)	13 (13,0)	0.90 (0.34 – 2.40)	1.000
Intraluminal lesion	36 (61)	43 (43,0)	2.07 (1.07 – 3.99)	0.042*

**Abbreviations: **SVCS, Superior vena cava syndrome.

*Statistically significant.

**Table 4:** Bivariate and multivariate logistic regression analysis of central squamous lung tumours.

**Variables**	**Bivariate Analysis**		**Multivariate Analysis**	
	**OR (95% CI)**	***p*-value**	**OR (95% CI)**	***p*-value**
Male	1.26 (0.61–2.59)	0.534	-	-
Elderly	1.62 (0.80–3.29)	0.181*	-	-
Smokers	1.16 (0.57–2.35)	0.673	-	-
Dyspnoea	1.17 (0.59–2.31)	0.643	-	-
Advanced Stage	0.79 (0.33–1.93)	0.618	-	-
SVCS	0.90 (0.34–2.40)	0.834	-	-
Intraluminal lesion	2.07 (1.07–3.99)	0.028*	2.07 (1.07–3.99)	0.028

**Abbreviations: **SVCS, Superior vena cava syndrome.

*Statistically significant.

## Data Availability

The data supporting the findings of the article is available from the corresponding author [M.E] on request.

## LIST OF ABBREVIATIONS

EBUS: Endobronchial Ultrasound
HE: Haematoxylin-Eosin
PCLT: Primary central lung tumours
SCC: Squamous Cell Carcinoma
SVCS: Superior Vena Cava Syndrome
TBNA: Transbronchial Needle Aspiration

## STANDARDS OF REPORTING

STROBE guidelines were followed.
